# Federated generalized additive models for location, scale and shape

**DOI:** 10.1186/s12874-025-02735-7

**Published:** 2025-12-09

**Authors:** Annika Swenne, Timm Intemann, Luis A. Moreno, Iris Pigeot

**Affiliations:** 1 Leibniz Institute for Prevention Research and Epidemiology - BIPS, Achterstraße 30, Bremen, 28359 Germany; 2 Faculty of Mathematics and Computer Science, University of Bremen, Post office box 330 440, Bremen, 28334 Germany; 3 GENUD (Growth, Exercise, Nutrition and Development) Research Group, University of Zaragoza, Pedro Cerbuna 12, Saragossa, 50009 Spain; 4 Centro de Investigación Biomédica en Red de Fisiopatología de La Obesidad y Nutrición (CIBERObn), Instituto de Salud Carlos III, Monforte de Lemos 3-5, Madrid, 28029 Spain; 5 Instituto Agroalimentario de Aragón (IA2), Calle Miguel Servet 177, Saragossa, 50013 Spain; 6 Instituto de Investigación Sanitaria Aragón (IIS Aragón), Avda. San Juan Bosco 13, Saragossa, 50009 Spain

**Keywords:** DataSHIELD, Federated analysis, Federated learning, GAMLSS, Growth curve, Percentile curve, Reference curve

## Abstract

**Background:**

The generalized additive model for location, scale and shape (GAMLSS) is a flexible regression model with a wide range of applications. In particular, it is the standard method to estimate age-specific percentile curves for clinical parameters for children and adolescents. Deriving international percentile curves requires large datasets that cover a diverse population. Such datasets are typically obtained by pooling data from multiple studies. However, due to ethical and legal constraints, physically sharing and pooling sensitive individual-level data might not always be permitted. Therefore, we aimed to develop a privacy-enhancing method to fit a GAMLSS.

**Methods:**

We developed a federated version of the GAMLSS algorithm which allows to co-analyze data from different sources, without physically transferring the data. Instead, data are analyzed locally within their secure home environments and only non-disclosive analysis results are shared. We implemented our method in DataSHIELD, an open-source software infrastructure for federated analysis in R, and investigated its theoretical properties. Considering two different use cases, we applied our algorithm to physically separated epidemiological study data and compared its results with the ones obtained by fitting a GAMLSS to the physically-pooled data. Furthermore, we evaluated the runtime of the federated GAMLSS against the original GAMLSS algorithm for varying number of observations and DataSHIELD servers.

**Results:**

We proved that, in theory, the federated GAMLSS yields identical results as the original GAMLSS algorithm, using the additivity of matrix multiplication in the fitting algorithm. Furthermore, we provided an implementation of the proposed algorithm and demonstrated that the federated GAMLSS implementation yielded the same results as the pooled GAMLSS in our examples, with only minor differences attributable to numerical computation. However, the runtime was more than 1000 times higher for fitting the federated compared to the pooled GAMLSS.

**Conclusions:**

In this paper, we propose a privacy-enhancing federated GAMLSS that yields virtually identical results as the original GAMLSS algorithm, without the need to physically pool the data.

## Background

The generalized additive model for location, scale and shape (GAMLSS) is a flexible regression framework to model the relationship between a single response variable and one or more explanatory variables [1]. Unlike classical linear regression with linear terms, where the relationship between the response and explanatory variable is assumed to be linear, the GAMLSS can additionally accommodate non-linear associations through non-parametric smoothing functions [2]. In addition, it supports the inclusion of random effects to account for clustering in the data, for example clustering within study centers. Whereas most regression models focus solely on one distribution parameter of the response variable, e.g. the mean (location parameter) in linear regression, the GAMLSS extends this by allowing the simultaneous modeling of further distribution parameters, like parameters for scale (e.g. variance) and shape (e.g. skewness and kurtosis) [2]. Finally, unlike other regression models, the GAMLSS offers more than 100 different distributions for the response variable.

Due to its flexibility, the GAMLSS has a wide range of applications, e.g. hydrological draught [3] or claim count analysis [4]. Furthermore, the GAMLSS is the standard method for estimating percentile curves, as it possesses several properties that make it particularly well suited for this task [5]. First, by assuming an underlying distribution, the GAMLSS allows precise estimation of extreme percentiles and enables direct calculation of z-scores and percentiles, which is not possible without distributional assumptions. Second, it naturally prevents crossing of the percentile curves, a problem that might occur with quantile regression. Finally, the GAMLSS can account for both skewness and kurtosis, which may be present in some measurements and need to be modeled to avoid distortion of the estimated percentiles.

Percentile curves are an essential reference in clinical routine to assess and monitor a child’s or adolescent’s health status. Reference curves have been constructed for various clinical parameters, e.g. child growth [6], physical activity [7] and ferritin and transferin concentration [8]. To establish percentile curves as an international reference, the underlying analysis dataset should not only be large but it should also cover a diverse population. This can for example be achieved by pooling data from multiple studies and study centers worldwide, as more and more done in recent studies [9–11]. These studies typically involve pooling individual-participant data from the different studies to derive the percentile curves.

**Fig. 1:**
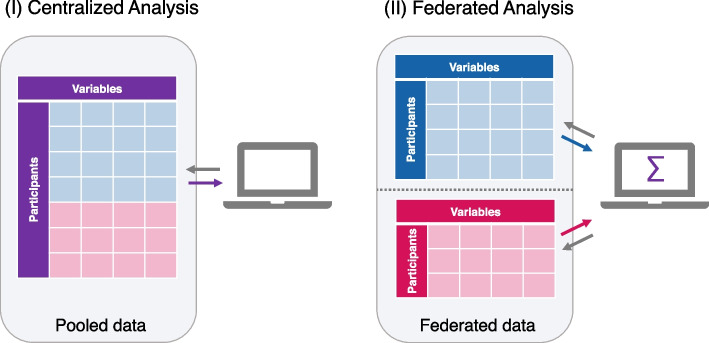
Centralized vs. federated analysis. In the centralized analysis (I) all individual-level data are pooled in a single file and analyzed jointly, whereas in the federated analysis (II) the data are stored separately and local analysis results from each data source are aggregated in the end. Each data source includes the same set of variables but for different participants

However, due to ethical and legal constraints, physically sharing and pooling sensitive individual-level data might not always be permitted. Federated analysis provides a solution for this problem by allowing data from different sources to be analyzed jointly, without physically sharing the data. Instead, data remain within their secure home environments and only non-disclosive analysis results are shared [12]. See Fig. 1 for a comparison of the federated analysis with the centralized analysis approach.

Different software platforms, that support federated analyses, have been developed. For example, DataSHIELD [13, 14] is an open-source software infrastructure for federated analysis in R [15] that is widely used in the biomedical sciences, e.g. in the ATHLETE [16], BioSHaRE [17] and NFDI4Health [18] project. Federated analysis platforms use special protocols to analyze federated data and for some statistical methods, including generalized linear models [19], maximum likelihood estimation and estimation of posterior distributions [20], federated versions are available that yield identical results as the analysis of physically-pooled individual-level data but without disclosing individual participants’ data. However, the implementation of some of these methods is still missing and for GAMLSS, federated versions were not available. Therefore, we aimed to develop and implement a federated version of the GAMLSS.

The contribution of this paper is threefold. First, we derive a federated version of the GAMLSS algorithm, and show that it yields identical results as the original algorithm with physically-pooled individual-level data (Federated generalized additive model for location, scale and shape section). Second, we provide the first implementation of the federated GAMLSS (Software implementation section) and demonstrate its application to physically separated epidemiological data from the IDEFICS/I.Family study [21, 22] for two different use cases (Linear regression example and Percentile curve example sections). And third, we compare the runtime of the federated approach with the runtime for fitting a GAMLSS to the same but physically-pooled data for different scenarios (Runtime comparison section).

## Methods

### DataSHIELD

**Fig. 2:**
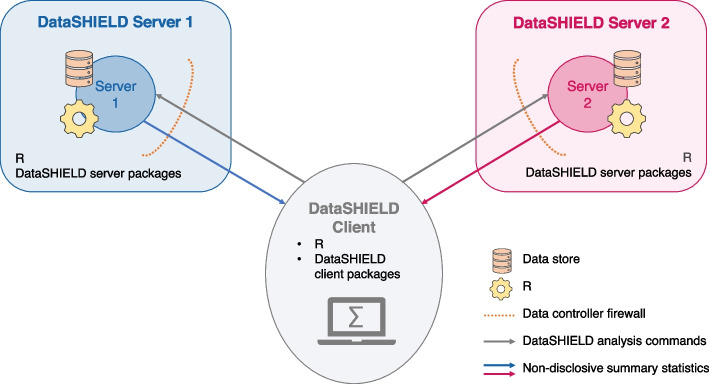
Illustration of the DataSHIELD infrastructure with two servers (adapted from [24]). The DataSHIELD servers, that store the individual-level data behind a firewall, are connected via one central analysis node, the DataSHIELD client. To analyze the data the client sends a request to each server, where the analysis is performed, and only non-disclosive summary statistics are returned to the client

DataSHIELD [13, 14] is a software infrastructure that enables federated analyses in R [15]. It follows a client-server architecture, in which the DataSHIELD servers - housing the individual-level data - remain with the respective data owners and are protected by their firewalls [14]. Figure 2 illustrates a DataSHIELD infrastructure with two DataSHIELD servers that are connected via one central analysis node, the DataSHIELD client. Each DataSHIELD server comprises the data warehouse Opal [23], a standard R server with the DataSHIELD server-side packages and an R parser that only allows DataSHIELD functions and their dependencies to run [14]. The DataSHIELD client also comprises a standard R server with the DataSHIELD client-side R packages, which are responsible for initiating DataSHIELD server-side functions.

To analyze data with DataSHIELD, the DataSHIELD client sends a request to run a specific R server-side function on the harmonized individual-level data, stored on the remote DataSHIELD servers [24]. If the analysis is permitted, low-dimensional non-disclosive summary statistics are returned to the DataSHIELD client. These summary statistics must adhere to the disclosure settings defined by the data owner, such as *k*-anonymity, i.e. only returning results that are based on at least *k* observations [25]. In principle, any R function can be implemented in DataSHIELD, as long as disclosure risks are eliminated [14]. In addition to one-step functions, which only call a single server-side function, it is also possible to perform multi-step or iterative functions, whereby server functions are called multiple times [14].

### Generalized additive model for location, scale and shape

The GAMLSS can be seen as a flexible extension of the classical linear regression model. It allows modeling not only the mean but also other parameters of the response variable’s distribution, e.g. its variance, using both parametric and non-parametric functions of the explanatory variables. The model is described in a general regression equation, which we now define formally.

Let $y_i$ denote the *i*-th observation of the response variable *Y*, that follows a distribution with probability density function $f_Y(y_i|\boldsymbol{\theta }_i)$, which is determined by the distribution parameter vector $\boldsymbol{\theta }_i^T = (\theta _{1i}, \theta _{2i}, \ldots , \theta _{pi})$ for $i=1,2,\ldots ,n$. Further, let $\boldsymbol{y}^T = (y_1, y_2, \ldots , y_n)$ be the vector of response variable observations and $\boldsymbol{\theta }_k^T = (\theta _{k1}, \theta _{k2}, \ldots , \theta _{kn})$ be the corresponding vector of distribution parameters for the *k*-th distribution parameter with $k=1,2,\ldots ,p$. Using a known monotonic link function $g_k$, the *k*-th distribution parameter can then be modeled via the *n*-dimensional predictor vector $\boldsymbol{\eta }_k$ as

1 $$\begin{aligned} g_k(\boldsymbol{\theta }_k) = \boldsymbol{\eta }_k = \boldsymbol{X}_k \boldsymbol{\beta }_k + \sum \limits _{j=1}^{J_k} \boldsymbol{Z}_{kj} \boldsymbol{\gamma }_{kj}, \end{aligned}$$

where $\boldsymbol{X}_k$ and $\boldsymbol{Z}_{kj}$ are $n \times J_k'$- and $n \times q_{kj}$-dimensional design matrices, $\boldsymbol{\beta }_k$ is a parameter vector of length $J_k'$ and $\boldsymbol{\gamma }_{kj} \sim \mathcal {N}_{q_{kj}}(\boldsymbol{0}, \boldsymbol{G}_{kj}(\boldsymbol{\lambda }_{kj})^{-1})$ is a $q_{kj}$-dimensional random variable with symmetric $q_{kj} \times q_{kj}$-dimensional matrix $\boldsymbol{G}_{kj}(\boldsymbol{\lambda }_{kj})$, which may depend on a vector of hyperparameters $\boldsymbol{\lambda }_{kj}$. To simplify the notation, we will refer to $\boldsymbol{G}_{kj}(\boldsymbol{\lambda }_{kj})$ as $\boldsymbol{G}_{kj}$ in this paper.

The first additive term in Eq. (1) allows to include parametric functions of the explanatory variables and the second additive term can be used to include random effect terms and nonparametric functions of the explanatory variables $h_{kj}(\boldsymbol{x}_{kj})$ via their basis representations with basis matrix $\boldsymbol{Z}_{kj}$ and corresponding parameter vector $\boldsymbol{\gamma }_{kj}$.

#### Model estimation

A GAMLSS is fitted by maximizing the penalized likelihood

2 $$\begin{aligned} l_p = \sum \limits _{i=1}^{n} \text {log}\left\{ f_Y(y_i|\boldsymbol{\theta }_i)\right\} - \frac{1}{2} \sum \limits _{k=1}^{p} \sum \limits _{j=1}^{J_k} \boldsymbol{\gamma }_{jk}^T \boldsymbol{G}_{kj} \boldsymbol{\gamma }_{jk} \end{aligned}$$

over $\boldsymbol{\beta }_k$ and $\boldsymbol{\gamma }_{kj}$ for fixed $\boldsymbol{\lambda }_{kj}$ [1]. To maximize the penalized likelihood for fixed $\boldsymbol{\lambda }_{kj}$, two algorithms, which both combine the Newton-Raphson and the backfitting algorithm, have been proposed: the Cole and Green algorithm (CG algorithm) [26] and the Rigby and Stasinopoulos algorithm (RS algorithm) [27]. The RS algorithm only uses the first and second derivatives of the likelihood function with respect to $\boldsymbol{\theta }_k$, whereas the CG algorithm also utilizes cross-derivatives. This paper focuses solely on the simpler RS algorithm, which is known to be more stable in practice ([28], p. 62).

The RS algorithm consists of three iterations that are nested inside each other: the outer iteration, that loops over the distribution parameters, the inner iteration, that estimates the *k*-th distribution parameter $\boldsymbol{\theta }_k$, or more precisely the corresponding predictor $\boldsymbol{\eta }_k$, and the backfitting iteration, that estimates the regression parameters $\boldsymbol{\beta }_k$ and $\boldsymbol{\gamma }_{kj}$ via (penalized) weighted least squares estimation.

If $\boldsymbol{\lambda }_{kj}$ is not fixed it can be estimated, either outside of the RS algorithm or CG algorithm (global approach) or within the backfitting algorithm (local approach) [29]. While the global methods can sometimes be more reliable, they are computationally expensive, whereas the local methods are much faster and often produce similar results compared to the global methods ([28], p. 78). See [29], for further details on the two approaches.

### Federated generalized additive model for location, scale and shape

**Fig. 3:**
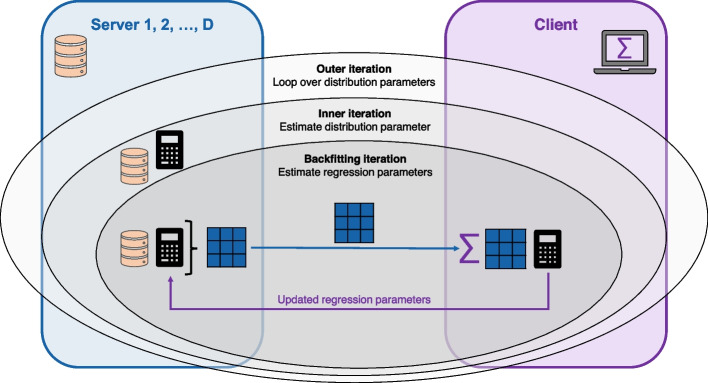
Illustration of the federated RS algorithm. The federated RS algorithm allows a GAMLSS to be fitted to data split across multiple servers. It consists of three iterations that are nested inside each other: the outer iteration, that loops over the distribution parameters, the inner iteration, that estimates the distribution parameter, and the backfitting iteration, that estimates the regression parameters. Within the inner and backfitting iteration, computations on the individual-level data are performed separately on each server. Low-dimensional matrices and vectors from each server are then aggregated on the client side during the backfitting iteration to update the estimated regression parameters. Each iteration is repeated until convergence

We propose the federated GAMLSS as a method to fit a GAMLSS to federated data. Figure 3 illustrates the federated RS algorithm for data that is split across *D* servers. It utilizes the fact that the computations on the individual-level data, that are required to perform the RS algorithm, can be performed separately on each server, and that only low-dimensional matrices and vectors must be shared with the client to update the regression parameters. More specifically, it exploits the additivity of the matrix multiplication across the servers. In Appendix A, we formally introduce the federated RS algorithm (Algorithm 1) and provide the technical proof that, by design, it yields identical results as the original RS algorithm.

Whether sharing the required matrices and vectors with the client poses a disclosure risk strongly depends on the structure of the design matrices. In particular, columns that are specific to a single individual or a very small group - such as categorical variables with sparse categories or random effects defined at the individual level - can be potentially disclosive. To safeguard participants’ privacy, such model specifications must be restricted in the federated GAMLSS implementation.

#### Software implementation

We implemented the federated GAMLSS using DataSHIELD [13, 14] through the client-side package dsGamlssClient [30] and the server-side package dsGamlss [31]. These packages enable fitting a federated GAMLSS with the client-side function ds.gamlss. After logging into the DataSHIELD server(s) with required data and the dsBase and dsGamlss packages installed, a GAMLSS can easily be fitted:
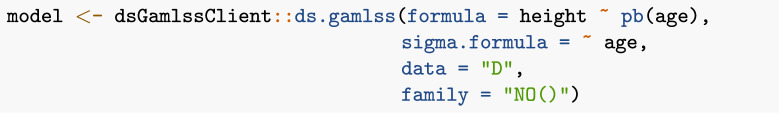


Currently, ds.gamlss supports fitting a federated GAMLSS with the normal, Box-Cox Cole and Green and Box-Cox power exponential distribution (BCPE distribution) using either parametric functions or P-splines (pb function) of the explanatory variables. Our federated implementation is based on the original gamlss function from the gamlss R package [1] but employs a different numerical approach for matrix inversion (Appendix A, Algorithm 1, lines 18 and 27), which may result in slightly different estimates. The structure of the output closely mirrors that of the original gamlss function, and the estimated regression parameters can be obtained in the same way via model$mu.coefficients. However, there are two key differences. First, individual-level data, such as residuals, are not returned. Second, the summary function is currently not implemented for ds.gamlss. As a result, it is not yet possible to obtain standard errors for the estimated regression coefficients.

After a federated GAMLSS has been fitted, the ds.predict.gamlss function can be used to predict the distribution parameters for new values of the explanatory variable:
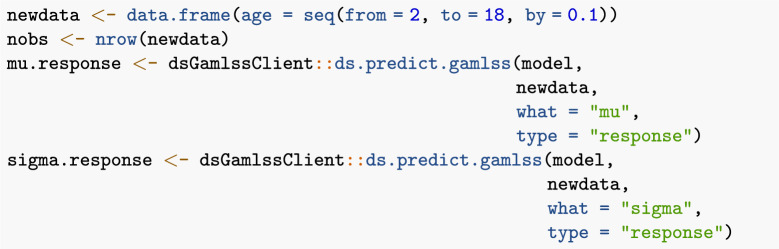


The estimated distribution parameters can then be used to estimate the percentiles using the appropriate quantile function from the gamlss.dist R package [32]:
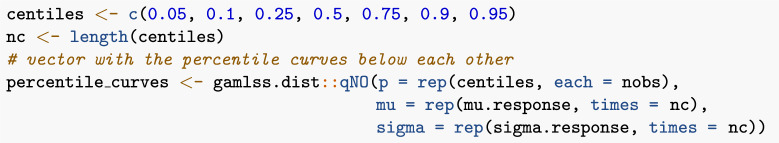


Finally, the estimated percentile curves may also be plotted for visualization:
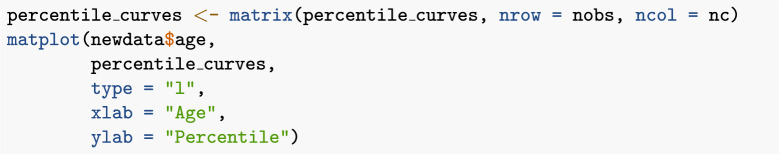


A complete working example demonstrating the use of the dsGamlssClient package, even without access to a DataSHIELD server, is available on GitHub [30].

Our federated GAMLSS implementation incorporates several disclosure control mechanisms. First, only low-dimensional non-disclosive results are returned to the client. Potentially identifying information like design matrices, fitted values and residuals are not returned to the client. Furthermore, the data owner can set various DataSHIELD privacy parameters to limit the disclosure risk. Fitting a federated GAMLSS is only permitted if the model’s degrees of freedom remain below a threshold specified by the data owner and the number of observations for each category of a categorical variable exceeds the corresponding threshold.

### Comparison federated vs. pooled approach

#### Example data

We used data from the IDEFICS/I.Family cohort (ISRCTN62310987) to compare our proposed federated GAMLSS with a GAMLSS that is applied to the same but physically-pooled data. The IDEFICS/I.Family cohort is a prospective multi-center study aimed at investigating the causes of diet- and lifestyle-related diseases in children and adolescents [21, 22]. The cohort comprises data collected at baseline from more than 16,000 children and adolescents from eight European countries (W1: 2007-2008) and during two follow-ups (W2: 2009-2010, W3: 2013-2014). The cohort study has been performed in accordance with the ethical standards as laid down in the 1964 Declaration of Helsinki and its later amendments or comparable ethical standards. Ethical approval was obtained from local ethic committees of each study center and parents provided written informed consent before their children were enrolled in the study. Examinations and sample collections were only conducted if both, parents and children, gave their consent. A detailed description of the IDEFICS/I.Family cohort can be found elsewhere [21, 22].

For our analyses, we used the participant’s sex, age (in years), height (in cm), the body mass index (BMI) (in $\text {kg}/\text {m}^2$) and the systolic blood pressure (SBP) (in mmHg). We only included cohort members from Germany and Spain. From these participants, only examination waves with the required measurements were considered, provided the participant was younger than 18 years at the time of the examination. To avoid bias from repeated measurements within the same individual, we included only one examination wave per participant. For participants with data from multiple examination waves, we prioritized measurements from wave W1, and, if unavailable, from wave W3, followed by wave W2. This approach maximized coverage across the full age range while ensuring that each participant contributed only once to the analysis.

#### Linear regression example

We used the example of modeling the SBP using BMI and sex as explanatory variables to demonstrate how the GAMLSS could be used to fit a linear regression model with different variances for the two countries. Such heteroscedastic models are often implicitly assumed in meta-analyses. We summarized the analysis dataset by sex, reporting the range, mean, and standard deviation of SBP and BMI. We compared the estimated regression parameters derived with the federated GAMLSS (ds.gamlss function) with the ones derived with the original gamlss function. For the federated analysis, data from Germany and Spain were stored in two separate datasets on the DataSHIELD server, whereas for the original gamlss function, the data were pooled in a single file. In both cases, we used the following model equation

$$\begin{aligned} \text {SBP}_i \sim \mathcal {N}(\mu _i, \sigma _i^2) & \\ \mu _i = \beta _{10} + \beta _{11}\text {sex}_i + \beta _{12}\text {BMI}_i & \\ log(\sigma _i) = \beta _{20} + \beta _{21}\text {country}_i, & \end{aligned}$$

which is similar to the classical linear model equation, with the only difference that it additionally allows different variances for the two countries.

#### Percentile curve example

For illustrative purposes, we derived age-specific height percentile curves for female participants from Germany and Spain. We calculated the range, mean and standard deviation of age and height to describe the analysis dataset. As in the example above, we compared the results derived with the federated GAMLSS (ds.gamlss function), in this case the age-specific height percentile curves, with the ones derived with the original gamlss function. For the federated analysis, age and height of the female participants from Germany and Spain were stored in two separate datasets on the DataSHIELD server, whereas for the original gamlss function, the data were pooled in a single file. In both cases, height was assumed to follow a BCPE distribution and the following model specifications were used

3 $$\begin{aligned} {\mathrm{height}}_i \sim BC\!P\!E(\mu _i, \sigma _i, \nu _i, \tau _i) & \nonumber \\ \mu _i = \beta _{10} + \beta _{11}\text {age}_i + h_{11}(\text {age}_i) & \nonumber \\ log(\sigma _i) = \beta _{20} + \beta _{21}\text {age}_i + h_{21}(\text {age}_i) & \nonumber \\ \nu _i = \beta _{30} + \beta _{31}\text {age}_i + h_{31}(\text {age}_i) & \nonumber \\ log(\tau _i) = \beta _{40} + \beta _{41}\text {age}_i + h_{41}(\text {age}_i), & \end{aligned}$$

where the four distribution parameters were modeled as a linear and nonparametric function of age using P-splines (via the pb function). Model fit was assessed via residual and worm plots. The fitted models were then used to derive age-specific 5, 10, 25, 50, 75, 90 and 95% percentiles using the quantile function of the BCPE distribution.

We considered two different sets of knots for the P-splines for the federated GAMLSS. First, we used the same knots as in the original GAMLSS applied to the physically-pooled data. However, usage of the same knots requires knowledge of the minimum and maximum of age, which might be disclosive. Therefore, we considered a second set of knots for the federated approach, that is determined by an anonymized minimum and maximum.

#### Runtime comparison

We compared the runtime of the DataSHIELD function ds.gamlss with the runtime of the original gamlss function for simulated data. Each runtime scenario was repeated 10 times with different simulated datasets.

Data were simulated as follows: First, two different GAMLSS, the first assuming a normal and the second a BCPE distribution, were fitted to the pooled German and Spanish data from the percentile curve example (Percentile curve example section). In both models, the distribution parameters were specified as nonparametric functions of age using P-splines (via the pb function). For the normal distribution we used the following model specification

$$\begin{aligned} \text {height}_i \sim \mathcal {N}(\mu _i, \sigma _i^2) & \\ \mu _i = \beta _{10} + \beta _{11}\text {age}_i + h_{11}(\text {age}_i) & \\ log(\sigma _i) = \beta _{20} + \beta _{21}\text {age}_i + h_{21}(\text {age}_i) & \end{aligned}$$

and for the BCPE distribution we used the same model specification (3) as for the percentile curve example (Percentile curve example section). The fitted models were then used to simulate height for a given age, based on the respective distribution and its estimated parameters. To simulate age, values were drawn from a uniform distribution between 2 and 18, rounded to one decimal digit to reflect the data in a real study.

To assess the runtime for a varying number of DataSHIELD servers, 2000 observations were simulated and split evenly into 1, 2, 4 and 8 datasets, which were then analyzed with the ds.gamlss function using the same model specification that was used to generate the data. The runtime for each setting was divided by the runtime of the original gamlss function, applied to the same but physically-pooled 2000 observations using the same model specification. Furthermore, we recorded the number of client-server communications that were needed to fit the federated GAMLSS. Note that we used the same knots for the P-splines for ds.gamlss and gamlss for the runtime analyses.

To assess the runtime for a varying number of observations, two DataSHIELD servers were mimicked, with 500, 1000 and 2000 observations on each server. Additionally, unevenly distributed numbers of observations across the servers were considered, with 500 observations on the first server and 2000 observations on the second server. Again, the runtime for ds.gamlss was divided by the runtime of the original gamlss function and the number of client-server communications was recorded. As above, we used the same knots for the P-splines for ds.gamlss and gamlss.

#### Computational environment

All analyses were conducted in R (version 4.4.2) [15]. The pooled analyses were performed on a 13th Gen Intel(R) Core(TM) i5-1335U with 1.30 GHz using the gamlss R package [1]. The federated analyses were performed with DataSHIELD [13, 14], using a DataSHIELD Opal server with R version 4.4.2 and 8 CPU cores (8 GHz and 32 GB memory) and the dsGamlssClient and dsGamlss packages. To simulate physically separated datasets on different DataSHIELD servers, separate connections were established within the local R session on the client, where each connection accessed a single dataset.

## Results

### Linear regression example

Data were available on 2437 participants (50% female) from Germany and 1628 (49% female) from Spain (Table 1). BMI and SBP were comparable for male and female participants and for Germany and Spain.

**Table 1:** Summary statistics for body mass index and systolic blood pressure by sex

Country	N	Body mass index (in kg/m^2^)				Systolic blood pressure (in mmHg)			
		Min	Max	Mean	SD	Min	Max	Mean	SD
*Female participants*									
Germany	1215	11.8	33.5	16.8	2.9	79.0	134.5	100.3	8.4
Spain	797	11.2	27.2	16.7	2.3	79.0	130.0	100.8	8.7
Pooled	2012	11.2	33.5	16.7	2.7	79.0	134.5	100.5	8.5
*Male participants*									
Germany	1222	12.3	33.0	16.4	2.4	72.0	149.5	100.5	8.4
Spain	831	12.4	29.0	16.6	2.3	80.0	147.5	101.8	8.9
Pooled	2053	12.3	33.0	16.5	2.3	72.0	149.5	101.0	8.6

Table 2 presents the regression parameters for the mean $\mu$ and standard deviation $\sigma$ of SBP, estimated using both the DataSHIELD ds.gamlss function and the original gamlss R function. The results indicate that the mean SBP was slightly lower in female participants compared to males and increased with BMI. Additionally, the standard deviation of SBP was higher for participants from Spain than from Germany. The table also demonstrates that the regression parameters estimated with the two functions differed only at the tenth or higher decimal places.

**Table 2:** Estimated regression parameters for systolic blood pressure

Distribution parameter	Regression parameter	Estimate	Decimal digit difference
$\mu$	Intercept $\beta _{10}$	80.0	10
$\mu$	Female $\beta _{11}$	−0.7	12
$\mu$	Body mass index $\beta _{12}$	1.3	12
$log(\sigma )$	Intercept $\beta _{20}$	2.0	13
$log(\sigma )$	Spain $\beta _{21}$	0.1	13

### Percentile curve example

Data were available on 1258 female participants from Germany and 806 female participants from Spain (Table 3). Ranges of age and height were slightly wider in Germany, compared to Spain. Furthermore, the mean age and height were higher for German compared to Spanish participants.

**Table 3:** Summary statistics for female participants' age and height

Country	N	Age (in years)				Height (in cm)			
		Min	Max	Mean	SD	Min	Max	Mean	SD
Germany	1258	2.0	17.9	7.0	3.0	82.6	186.0	122.6	17.5
Spain	806	2.3	15.0	6.1	2.1	82.3	171.0	115.7	14.2
Pooled	2064	2.0	17.9	6.7	2.7	82.3	186.0	119.9	16.7

**Fig. 4:**
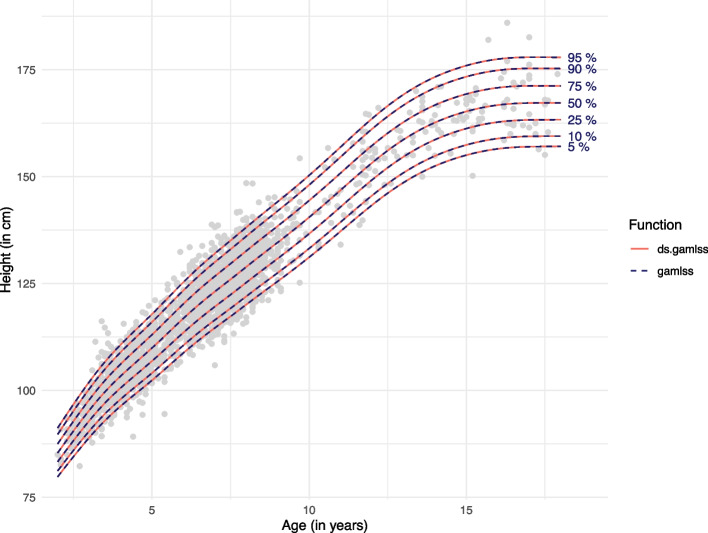
Estimated age-specific height percentile curves: federated vs. pooled approach. The age-specific height percentile curves fitted to the federated data (ds.gamlss function) are virtually identical to the ones obtained by fitting a model to the same, but physically-pooled data (gamlss function)

Figure 4 shows no difference between the age-specific height percentile curves, estimated with the DataSHIELD function ds.gamlss and those fitted to the same but physically-pooled data, via the gamlss function. This was the case for both sets of knots for ds.gamlss. Furthermore, if the same knots were used for ds.gamlss and gamlss both functions yielded the same estimated age-specific distribution parameters, up to the third decimal place (Appendix B, Fig. 7). If the anonymized minimum and maximum were used to determine the knots for ds.gamlss, the estimated age-specific distribution parameters differed slightly for one parameter, as shown in Fig. 8 (Appendix B). Residual and worm plots for the fitted model are provided in Appendix C (Figs. 9 and 10).

### Runtime comparison

**Fig. 5:**
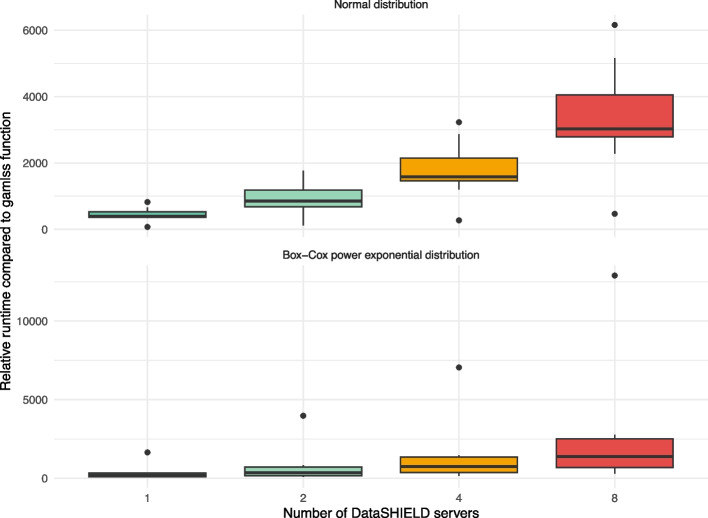
Relative runtime compared to the gamlss function for different numbers of DataSHIELD servers. The box plots show the increase in runtime for fitting a GAMLSS with DataSHIELD relative to a GAMLSS that has been fitted to the same, but physically-pooled data, for different numbers of DataSHIELD servers. For both, the normal and BCPE distribution, the relative runtime increases with the number of DataSHIELD servers

**Fig. 6:**
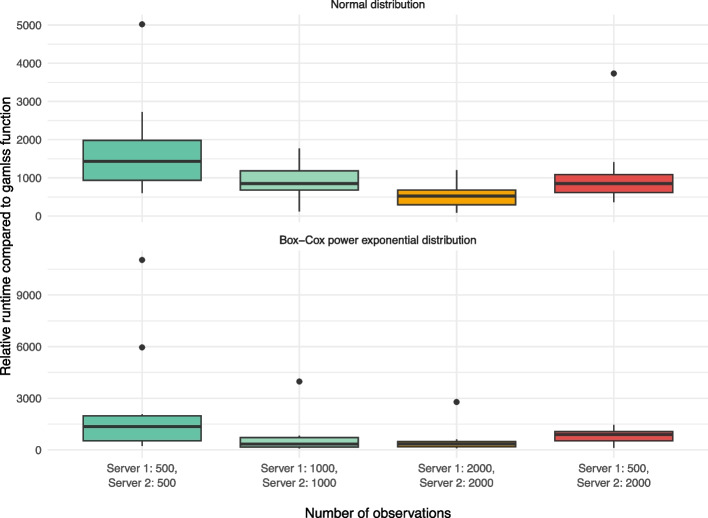
Relative runtime compared to the gamlss function for different sample sizes. The box plots show the increase in runtime for fitting a GAMLSS with DataSHIELD relative to a GAMLSS that has been fitted to the same but physically-pooled data, for two DataSHIELD servers with different sample sizes. For both, the normal and BCPE distribution, the relative runtime tends to decrease with increasing sample size

**Fig. 7:**
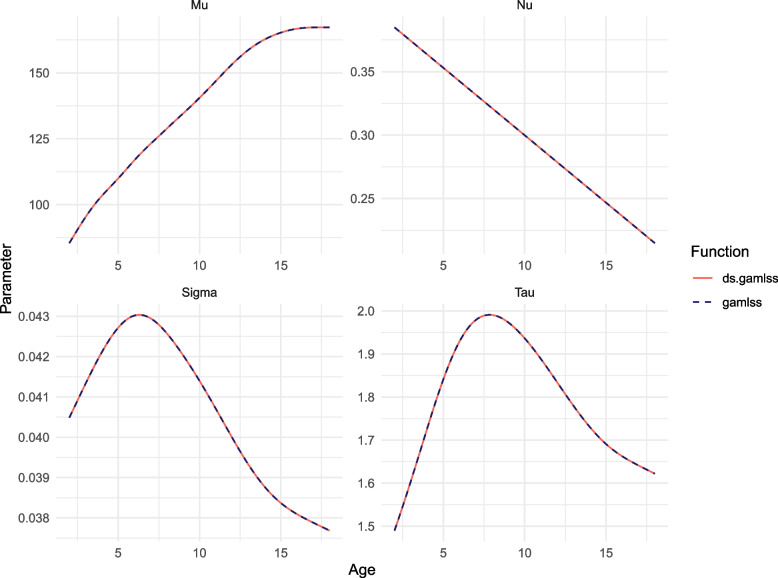
Estimated distribution parameters depending on age: federated vs. pooled approach with the same knots. If the same knots are used for the P-splines in the federated (ds.gamlss function) and pooled approach (gamlss function), both approaches yield virtually identical results for the four distribution parameters $\mu , \sigma , \nu , \tau$ of the BCPE distribution for height, conditional on age

**Fig. 8:**
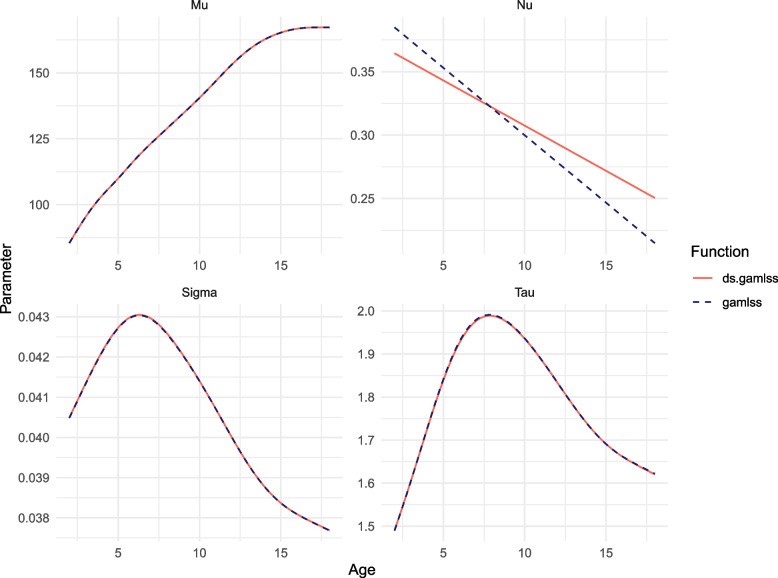
Estimated distribution parameters depending on age: federated vs. pooled approach with different knots. If different knots are used for the P-splines in the federated (ds.gamlss function) and pooled approach (gamlss function), the estimated four distribution parameters $\mu , \sigma , \nu , \tau$ of the BCPE distribution for height, conditional on age, differ slightly, as it is the case for $\nu$ in this example

**Fig. 9:**
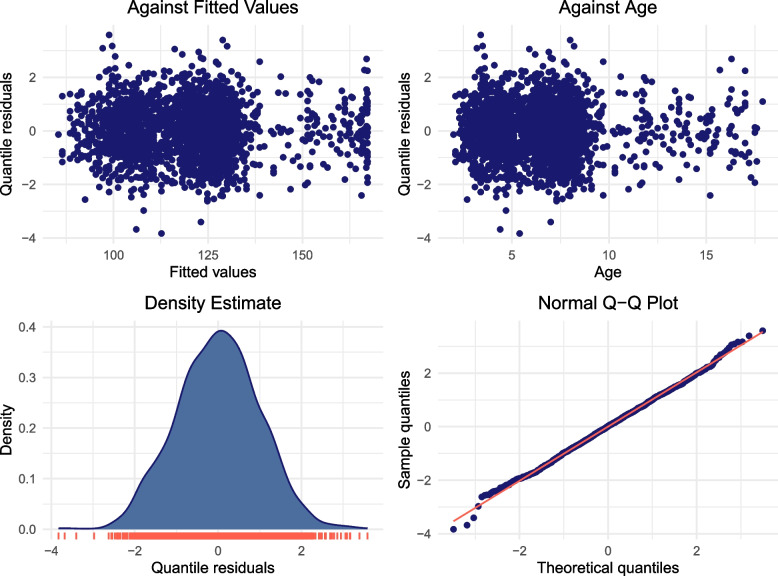
Residual plots for the fitted GAMLSS. The quantile residuals plotted against the fitted values (top left corner) and against age (top right corner) are randomly scattered around the horizontal line at zero. The estimated density of the residuals (bottom left corner) and the normal Q-Q plot (bottom right corner) show that the residuals are approximately normally distributed

**Fig. 10:**
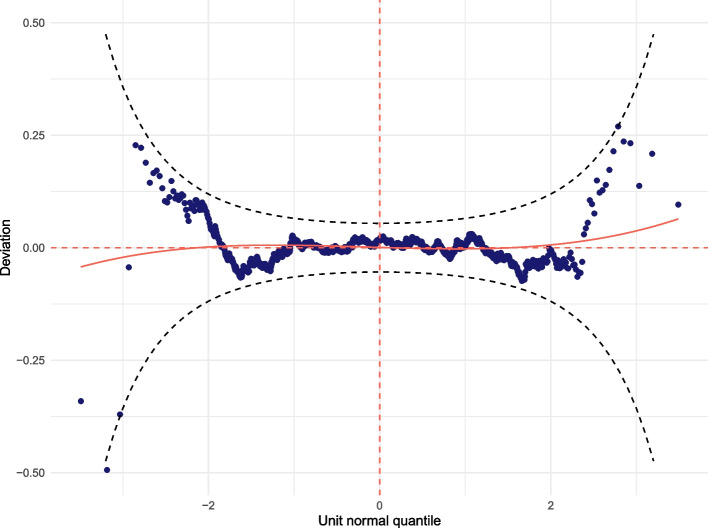
Worm plot for the fitted GAMLSS. The worm plot shows that the normalized quantile residuals are approximately normally distributed, since the fitted curve (red line) is close to the horizontal line and only few observations fall outside the acceptance range (dashed black line)

**Fig. 11:**
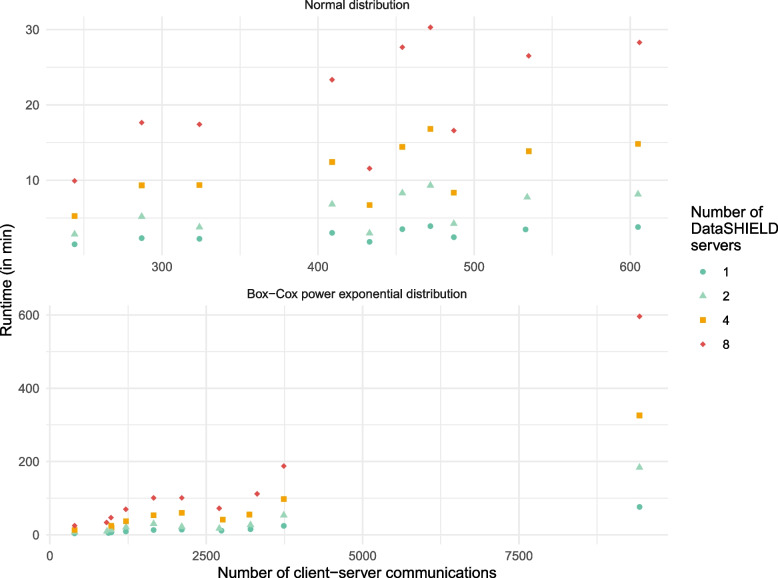
Runtime over the number of client-server communications for different numbers of DataSHIELD servers. The scatter plot shows that the runtime to fit a federated GAMLSS increases with the number of DataSHIELD servers and the number of client-server communications

**Fig. 12:**
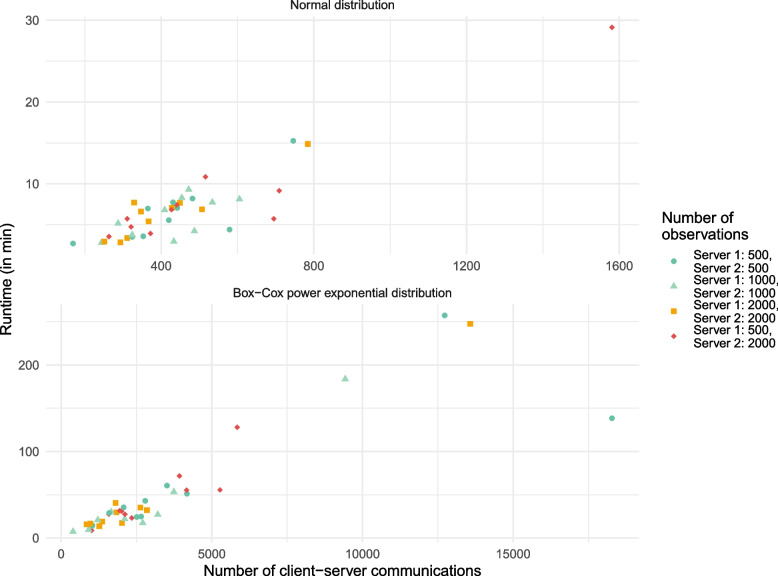
Runtime over the number of client-server communications for different sample sizes. The scatter plot shows that the runtime to fit a federated GAMLSS increases with the number of client-server communications

Figure 5 shows the runtime for fitting a GAMLSS with the DataSHIELD function ds.gamlss for different numbers of DataSHIELD servers. The figure shows that the runtime is more than thousand times higher for the ds.gamlss compared to the gamlss function. This applies to both distributions. Furthermore, the runtime increased with the number of DataSHIELD servers. If different sample sizes were used to fit the GAMLSS with DataSHIELD, as shown in Fig. 6 for the example of two DataSHIELD servers, the relative runtime tended to decrease with the number of observations. For both, different numbers of DataSHIELD servers and sample sizes, the runtime was strongly dependent on the number of client-server communications (Appendix D, Figs. 11 and 12). In some cases, more than 1,000 communications were required, depending on the number of parameters estimated.

## Discussion

We developed a federated version of the GAMLSS and proved that, in theory, it yields identical results as the analysis of physically-pooled individual-level data. Additionally, we provided a first implementation of the federated GAMLSS and demonstrated its potential by estimating age-specific height percentile curves from physically separated epidemiological study data. To the best of our knowledge, this is the first paper to describe and implement a federated GAMLSS. The proposed federated GAMLSS is a flexible extension of the federated generalized linear model [19]. Both models exploit the additivity of the matrix multiplication across servers. The ability to obtain virtually identical results with the federated GAMLSS as with the analysis of physically-pooled data, while protecting the study participants’ privacy, is a particular strength of our proposed federated GAMLSS. Minor differences between the federated and pooled estimates are likely due to different numerical approaches for matrix inversion in the implementations.

Nonetheless, there are some limitations. Most importantly, the runtime was markedly increased for the federated compared to the pooled approach. Our analyses suggest that the increase in runtime was mainly due to the high communication demand for fitting a federated GAMLSS, which cannot be easily mitigated by increasing the computational capacities. In each backfitting iteration, the federated GAMLSS algorithm has to wait for responses from all DataSHIELD servers, before proceeding to the next step. Thus, the increase in runtime could be even worse when one or more data providers have a particularly slow connection. To reduce the communication demand, we propose performing a certain number of backfitting iterations separately on each server before sharing the updates with the client. This allows the server to perform multiple iterations of model fitting without the need to communicate with the client after each iteration. However, further research is needed to decide when this approach converges and to determine the optimal number of iterations that balances model fitting accuracy and runtime.

We used DataSHIELD to implement the federated GAMLSS, given its widespread use in biomedical research. We acknowledge that it can be challenging for the data providers to set up and maintain the DataSHIELD servers. Furthermore, data harmonization can be more demanding within the DataSHIELD infrastructure, since the harmonization must be coordinated across all data providers and must be performed ahead of time [13]. Nonetheless, these challenges regarding the DataSHIELD implementation do not limit the applicability of the federated GAMLSS algorithm itself, since it could also be implemented in any other federated analysis platform.

The federated GAMLSS enhances privacy by design and via the DataSHIELD non-disclosure mechanisms [25]. However, despite the rigorous disclosure control, privacy can never be completely guaranteed. In particular, there might be external information available that could be used in combination with the results from the federated GAMLSS to infer sensitive personal information [33]. Furthermore, it is possible that analyses previously regarded as non-disclosive may unexpectedly turn out to be disclosive [19]. For example, it has been shown recently that the ability to create known linearly independent vectors on the client-side and functions to compute sample means and covariances in federated analysis platforms could be exploited to infer individual-level data [34]. If users identify potential disclosure risks, they are encouraged to report them such that appropriate actions can be taken to block the loop-holes.

We provided an implementation for the important case of a semi-parametric GAMLSS with P-splines. Future research could extend the federated GAMLSS implementation in several directions, such as incorporating additional distributions for the response variable, handling time-to-event outcomes, integrating random effect terms, exploring other types of spline functions, or even expand the federated GAMLSS to federated Bayesian additive models for location, scale and shape [35]. The development of model diagnostic tools would also further enhance the applicability of the federated GAMLSS implementation. Additionally, statistical inference, such as the computation of standard errors or confidence intervals for estimated parameters, is currently not implemented and represents an important avenue for future work. Such extensions, however, require careful consideration of potential disclosure risks and appropriate mitigation strategies. For instance, subject-specific random effects might pose a risk of re-identification and should be handled carefully, if not prohibited [19]. The same also holds for the residuals, which are required for most model diagnostics [19]. Mitigation strategies for the latter could be the use of privacy-preserving data visualizations for the residuals [33].

## Conclusions

GAMLSS is a popular regression model that is used to estimate percentile curves. To obtain generalizable and meaningful results, large and representative analysis datasets are required, which can be obtained by pooling data from multiple studies. However, due to ethical and legal constraints, physically sharing and pooling individual-level data might not always be permitted. Thus, privacy-preserving approaches, like federated analyses, are needed. In this paper, we developed a privacy-enhancing federated GAMLSS and proved that, in theory, it yields identical results as the original GAMLSS algorithm, without the need to physically pool the data. We further implemented the federated algorithm and showed that, in practice, it yields virtually identical results as the pooled GAMLSS. This is an important step toward enabling research on larger, more diverse datasets while protecting the privacy of the participants.

## Figures and Tables

**Figure:**
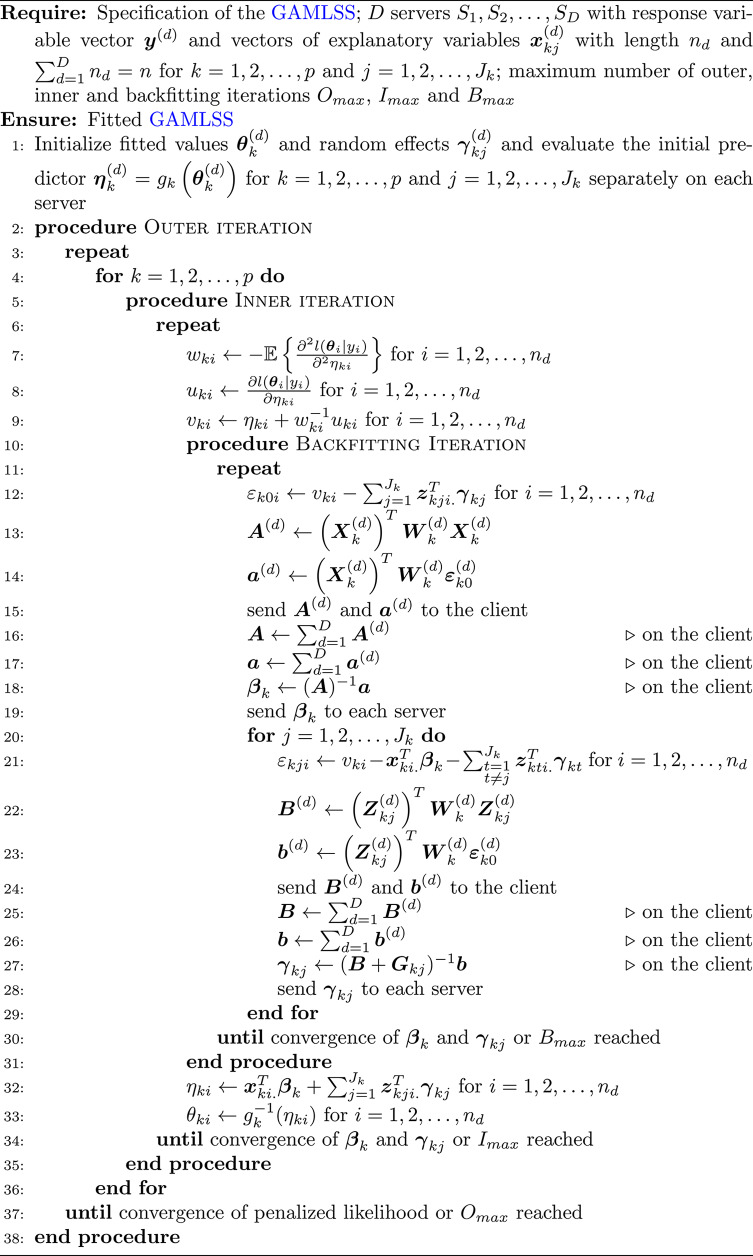
**Algorithm 1** The federated Rigby and Stasinopoulos algorithm

**Figure:**
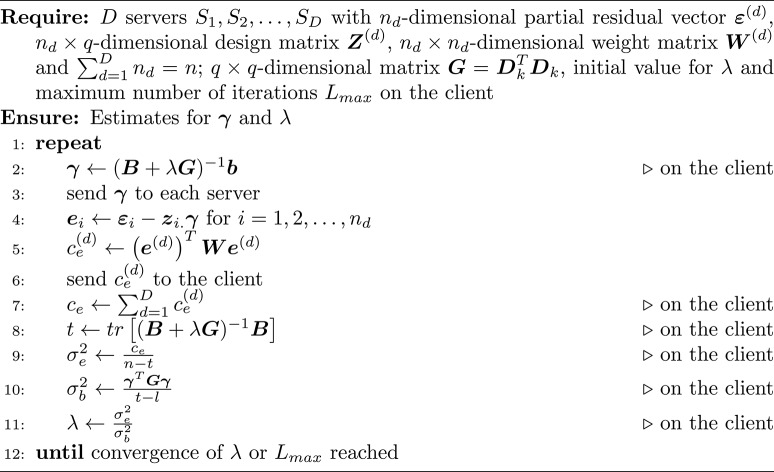
**Algorithm 2** The federated local maximum-likelihood estimation

## Data Availability

Due to the prospective nature of the ongoing IDEFICS/I.Family cohort study, the full anonymization of study data is ruled out and use of data requires a mutual agreement between our study consortium and interested third parties on a case-by-case basis. For corresponding requests, please contact the study coordinator (ahrens@leibniz-bips.de) or apply via the BIPS Research Data Portal (https://www.bips-institut.de/en/research/research-data-portal.html).

## Appendix A: Federated algorithms

### Federated Rigby and Stasinopoulos algorithm

Algorithm 1 shows the federated version of the federated Rigby and Stasinopoulos algorithm for data that are split across *D* servers $S_1, S_2, \ldots , S_D$ with $n_d$ observations on each server and $\sum \nolimits _{d=1}^{D} n_d = n$. This implies, that also the design matrices $\boldsymbol{X}_k$ and $\boldsymbol{Z}_{kj}$, the partial residuals $\boldsymbol{\varepsilon }_{kj^*}$ with $j^*=0,1,2,\ldots ,J$ and the weight matrix $\boldsymbol{W}_k$ are split across the servers, i.e.

$$\begin{aligned} \boldsymbol{X}_k & = \left[ \begin{array}{c} \boldsymbol{X}_k^{(1)} \\ \boldsymbol{X}_k^{(2)} \\ \vdots \\ \boldsymbol{X}_k^{(D)} \end{array}\right] , \boldsymbol{Z}_{kj} = \left[ \begin{array}{c} \boldsymbol{Z}_{kj}^{(1)} \\ \boldsymbol{Z}_{kj}^{(2)} \\ \vdots \\ \boldsymbol{Z}_{kj}^{(D)} \end{array}\right] ,\\ \boldsymbol{\varepsilon }_{kj^*} & = \left( \begin{array}{c} \boldsymbol{\varepsilon }_{kj^*}^{(1)} \\ \boldsymbol{\varepsilon }_{kj^*}^{(2)} \\ \vdots \\ \boldsymbol{\varepsilon }_{kj^*}^{(D)} \end{array}\right) \ \text {and}\ \boldsymbol{W}_k = \text {diag} \left( \begin{array}{c} \boldsymbol{w}_{k}^{(1)} \\ \boldsymbol{w}_{k}^{(2)} \\ \vdots \\ \boldsymbol{w}_{k}^{(D)} \end{array}\right) , \end{aligned}$$

with $n_d \times J'$-dimensional design matrix $\boldsymbol{X}_k^{(d)}$, $n_d \times q_j$-dimensional design matrix $\boldsymbol{Z}_{kj}^{(d)}$, $n_d$-dimensional partial residual vector $\boldsymbol{\varepsilon }_{kj^*}^{(d)}$ and $n_d$-dimensional weight vector $\boldsymbol{w}_k^{(d)}$ for server $S_d$ with $d=1,2,\ldots ,D$. Throughout this appendix, we will use $\boldsymbol{x}_{ki.}$ and $\boldsymbol{z}_{kji.}$ to refer to the *i*-th row vector from the design matrices $\boldsymbol{X}_{k}$ and $\boldsymbol{Z}_{kj}$, respectively.

Since the weight $w_{ki}$, the score $u_{ki}$ and the working variable $v_{ki}$ in the inner iteration contain only individual-level data from the *i*-th participant (Algorithm 1, lines 7–9), they can be computed separately for each participant, and hence they can be computed separately on the servers. The same also holds for the partial residuals $\varepsilon _{kj^*i}$ in the backfitting iteration (Algorithm 1, lines 12 and 21).

To compute the weighted least squares estimator (WLSE) $\boldsymbol{\beta }_k$ (Algorithm 1, line 18) and the penalized weighted least squares estimator (PWLSE) $\boldsymbol{\gamma }_{kj}$ (Algorithm 1, line 27), we use that the matrix multiplication is additive across the servers, i.e.

$$\begin{aligned} \boldsymbol{X}_k^T \boldsymbol{W}_k \boldsymbol{X}_k = \sum \limits _{d=1}^{D} \left( \boldsymbol{X}_k^{(d)}\right) ^T\boldsymbol{W}_k^{(d)}\boldsymbol{X}_k^{(d)} & = \sum \limits _{d=1}^{D} \boldsymbol{A}^{(d)}\\ \boldsymbol{Z}_{kj}^T \boldsymbol{W}_k \boldsymbol{Z}_{kj} = \sum \limits _{d=1}^{D} \left( \boldsymbol{Z}_{kj}^{(d)}\right) ^T\boldsymbol{W}_k^{(d)}\boldsymbol{Z}_{kj}^{(d)} & = \sum \limits _{d=1}^{D} \boldsymbol{B}^{(d)}\\ \boldsymbol{X}_k^T \boldsymbol{W}_k \boldsymbol{\varepsilon }_{k0} = \sum \limits _{d=1}^{D} \left( \boldsymbol{X}_k^{(d)}\right) ^T\boldsymbol{W}_k^{(d)}\boldsymbol{\varepsilon }_{k0}^{(d)} & = \sum \limits _{d=1}^{D} \boldsymbol{a}^{(d)}\\ \boldsymbol{Z}_{kj}^T \boldsymbol{W}_k \boldsymbol{\varepsilon }_{kj} = \sum \limits _{d=1}^{D} \left( \boldsymbol{Z}_{kj}^{(d)}\right) ^T\boldsymbol{W}_k^{(d)}\boldsymbol{\varepsilon }_{kj}^{(d)} & = \sum \limits _{d=1}^{D} \boldsymbol{b}^{(d)}. \end{aligned}$$

This implies that each server can return the $J' \times J'$- and $q_j \times q_j$-dimensional matrices $\boldsymbol{A}^{(d)}$ and $\boldsymbol{B}^{(d)}$ and the $J'$- and $q_j$-dimensional vectors $\boldsymbol{a}^{(d)}$ and $\boldsymbol{b}^{(d)}$ to the client, which aggregates them to compute the WLSE and PWLSE. As a result, the federated RS algorithm yields identical results as the original RS algorithm, without the need to physically pool the data.

To estimate $\boldsymbol{\lambda }_{kj}$ for the PWLSE, we used the local likelihood-based approach [29]. Appendix the following section provides its federated version (Algorithm 2) and shows that it yields identical results as the pooled approach.

### Federated algorithm for smoothing parameter estimation

We provide a federated version of the local likelihood-based approach [29] to estimate $\boldsymbol{\lambda }_{kj}$ within the federated RS algorithm (Appendix Federated RS algorithm, Algorithm 1) for the special case of $\boldsymbol{G}_{kj}(\boldsymbol{\lambda }_{kj}) = \lambda _{kj}\boldsymbol{G}_{kj} = \lambda _{kj}\boldsymbol{D}_l^T\boldsymbol{D}_l$ with $(q_j-l) \times q_j$-dimensional difference matrix $\boldsymbol{D}_l$ of order *l*. For $l=0$, the difference matrix reduces to the identity matrix, i.e. $\boldsymbol{D}_l = \boldsymbol{I}_{q_j}$, which corresponds to the standard independent random effects model.

To estimate $\lambda _{kj}$, the federated local maximum-likelihood estimation (Algorithm 2) is applied within each backfitting iteration (Appendix Federated RS algorithm, Algorithm 1, line 27) to the partial residuals $\boldsymbol{\varepsilon }_{kj}$ for $j=1,2,...,J_k$ for the *k*-th distribution parameter $\boldsymbol{\theta }_k$. In the federated approach, the partial residuals $\boldsymbol{\varepsilon }_{kj}$ with $j=1,2,...,J_k$, the design matrices $\boldsymbol{Z}_{kj}$ and the weight matrices $\boldsymbol{W}_k$ are split across *D* servers $S_1, S_2, \ldots , S_D$, i.e.

$$\begin{aligned} \boldsymbol{\varepsilon }_{kj} & = \left( \begin{array}{c} \boldsymbol{\varepsilon }_{kj}^{(1)} \\ \boldsymbol{\varepsilon }_{kj}^{(2)} \\ \vdots \\ \boldsymbol{\varepsilon }_{kj}^{(D)} \end{array}\right) , \boldsymbol{Z}_{kj} = \left[ \begin{array}{c} \boldsymbol{Z}_{kj}^{(1)} \\ \boldsymbol{Z}_{kj}^{(2)} \\ \vdots \\ \boldsymbol{Z}_{kj}^{(D)} \end{array}\right] ,\ \text {and}\\ \boldsymbol{W}_k & = \text {diag} \left( \begin{array}{c} \boldsymbol{w}_{k}^{(1)} \\ \boldsymbol{w}_{k}^{(2)} \\ \vdots \\ \boldsymbol{w}_{k}^{(D)} \end{array}\right) , \end{aligned}$$

with $n_d$-dimensional partial residual vector $\boldsymbol{\varepsilon }_{kj}^{(d)}$, $n_d \times q_j$-dimensional design matrix $\boldsymbol{Z}_{kj}^{(d)}$ and $n_d$-dimensional weight vector $\boldsymbol{w}_k^{(d)}$ for server $d=1,2,\ldots ,D$ with $\sum \nolimits _{d=1}^{D} n_d = n$. Note that for notational convenience, the indices *k* and *j* are omitted from Algorithm 2 and in the remainder of this appendix. Further, we use $\boldsymbol{z}_{i.}$ to denote the *i*-th row vector from the design matrix $\boldsymbol{Z}$.

In the federated local maximum-likelihood estimation, the residuals $\boldsymbol{e}_i$ are computed separately on each server, since they only contain the PWLSE $\boldsymbol{\gamma }$ and individual-level data from the *i*-th participant (Algorithm 2, line 4). To compute the variance component $c_e$ (Algorithm 2, line 7), we use that the matrix multiplication is additive across the servers, i.e.

$$\begin{aligned} \boldsymbol{e}^T\boldsymbol{We} = \sum \limits _{d=1}^D \left( \boldsymbol{e}^{(d)}\right) ^T\boldsymbol{W}^{(d)}\boldsymbol{e}^{(d)} = \sum \limits _{d=1}^D c_e^{(d)}. \end{aligned}$$

This implies that each server can return its local value $c_e^{(d)}$ to the client, which then aggregates these values to compute the overall variance component. The other variance components can be computed directly on the client to obtain the variances $\sigma _e^2$ and $\sigma _b^2$ (Algorithm 2, lines 9 and 10) to update $\lambda$. Accordingly, the federated local maximum-likelihood estimation yields identical results as the original local maximum-likelihood estimation, without the need to physically pool the data.

## Appendix B: Comparison of estimated distribution parameters for the percentile curve example

This appendix compares the estimated age-specific distribution parameters for the federated (ds.gamlss function) and the pooled approach (gamlss function) for the percentile curve example. Figure 7 shows the results when the same knots were used for the P-splines in both functions and Fig. 8 shows the results when the knots for the ds.gamlss function were determined by an anonymized minimum and maximum.

## Appendix C: Model diagnostics for the percentile curve example

This appendix provides the model diagnostics for the fitted GAMLSS for the percentile curve example. Figure 9 shows the residual plots and Fig. 10 shows the worm plot.

## Appendix D: Relationship between runtime and number of client-server communications

This appendix contains scatter plots of the runtime for fitting a federated GAMLSS over the number of client-server communications. Figure 11 shows the plot for varying number of DataSHIELD servers and Fig. 12 shows the plot for varying number of observations.

## Abbreviations

BCPE distribution: Box-Cox power exponential distribution
BMI: Body mass index
CG algorithm: Cole and Green algorithm
GAMLSS: Generalized additive model for location, scale and shape
PWLSE: Penalized weighted least squares estimator
RS algorithm: Rigby and Stasinopoulos algorithm
SBP: Systolic blood pressure
WLSE: Weighted least squares estimator
